# Numerical Simulation of Flow Characteristics for Supercritical CO_2_-Sprayed Polyurethane Resin

**DOI:** 10.3390/polym16070940

**Published:** 2024-03-29

**Authors:** Chichao Li, Chengrui Zhang, Minghua Xiang, Qing Chen, Zhenyang Luo, Yanlong Luo

**Affiliations:** 1 College of Science, Nanjing Forestry University, Nanjing 210037, China; l885661983@gmail.com (C.L.); zcr19850410054@163.com (C.Z.); luozhenyang@njfu.edu.cn (Z.L.); 2 Shaoxing Huachuang Polyurethane Co., Ltd., Shaoxing 312037, China; minghuaxiang2024@163.com; 3 College of Mechanical and Electronic Engineering, Nanjing Forestry University, Nanjing 210037, China; qchen@njfu.edu.cn

**Keywords:** volatile organic compounds, supercritical CO_2_, polyurethane, computational fluid dynamics

## Abstract

Conventional paint spraying processes often use small molecule organic solvents and emit a large amount of volatile organic compounds (VOCs) that are highly toxic, flammable, and explosive. Alternatively, the spraying technology using supercritical CO_2_ (scCO_2_) as a solvent has attracted attention because of its ability to reduce VOC emissions, but the flow characteristics of coatings have not been thoroughly studied. Therefore, we numerically simulate the spraying process based on the actual process of scCO_2_ spraying polyurethane coatings by computational fluid dynamics (CFD). The effects of inlet pressure and volume fraction of scCO_2_ on the fluid motion parameters inside the nozzle as well as the atomization effect of droplets outside the nozzle are investigated. The simulated results show that a fluid with a large volume fraction of scCO_2_ will obtain a smaller density, resulting in a larger velocity and a larger distance for the spray to effectively spray. Higher coating content and bigger inlet pressures will result in higher discrete phase model (DPM) concentrations, and thus a bigger inlet pressure should be used to make the droplets more uniform across the 30° spray range. This study can provide theoretical guidance for the process of scCO_2_-sprayed polyurethane resin.

## 1. Introduction

Polyurethane (PU) is one of the most versatile high-performance polymers used in coatings, adhesives, elastomers, foams, composites, and other processable materials [1,2]. Polyurethane coatings have excellent chemical resistance, mechanical/physical durability, and heat resistance [3,4,5,6]. PU are amongst the most advantageous materials in the coatings industry and are used in a variety of sectors due to their exceptional adherence to a wide range of surfaces, fast drying time, chemical resistance, and low flammability [7]. The research on polyurethane coatings has been intensive, such as on the anti-icing behavior of polyurethane coatings [8]; self-cleaning, anti-graffiti, and corrosion-resistant functional UV-cured waterborne polyurethane coatings [9]; self-repairable polyurethane coatings, [10], etc. Currently, the commonly used spraying processes include flame spraying, high-velocity oxygen fuel (HVOF)/high-velocity air fuel (HVAF), plasma spraying roller coating, air spraying, electrostatic spraying, electrophoretic spraying, high-pressure airless spraying, etc. [11,12]. Polyurethane coatings are generally applied by high-pressure airless spraying, that is, directly pressurizing the paint to form a high-pressure fluid, and then the muzzle forms atomized droplets acting on the surface of the object (wall or wood surface).

Conventional spraying processes often use small molecular organic compounds as solvents (such as xylene, cyclohexanone, or butanone, etc.), so the spraying process will emit a large number of volatile organic compounds (VOCs), which are flammable, explosive, and toxic. VOCs are very harmful to human health and the environment and have been called the “invisible killer” of human beings. How to reduce the emission of VOCs has also become a new development goal for the coating industry. The use of supercritical carbon dioxide (scCO_2_) as an alternative to traditional aqueous and organic solvents is a route to solve the problem [13]. Because scCO_2_ with a critical point of 73.9 bar and 31 °C has gas–liquid dual properties, it has good solubility and diffusion properties for polymer resins and is non-toxic and harmless [14,15,16]. Furthermore, the low cost, low viscosity, stability, and low toxicity of scCO_2_ make it one of the best candidates to replace organic solvents [17]. scCO_2_ as a coating solvent has many significant advantages: it reduces the use of diluents, reducing VOCs from the source; the VOC reduction of scCO_2_ coating technology is not as good as waterborne coatings, but the drying energy consumption is only about one-twentieth of that of the waterborne coating; scCO_2_ coating technology requires a small amount of air volume, and thus can significantly improve the efficiency of the coating; scCO_2_ does not change the composition of the coatings and the color, and thus can ensure that the quality and appearance of the coating is no different from that of the traditional solvent-based coating. When scCO_2_ dioxide is used instead of organic solvents in spraying, compounds with sufficiently low surface energies are dissolved in scCO_2_. During this process, the mixture is expanded through a nozzle to form a coating on the surface through a technique called rapid expansion of supercritical solution (RESS) [18]. Therefore, the spraying technology based on scCO_2_ has attracted much attention. 

In recent years, many researchers have paid great attention to computational fluid dynamics (CFD) and studied the atomization behavior of fluids. For example, scCO_2_ and alkyl ketene dimer (AKD) mixed coatings were successfully coated on several different substrates such as glass, aluminum, paper, polyethylene terephthalate (PET), and poly-tetrafluoroethylene (PTFE), and the superhydrophobic properties of the coatings were studied the optimal spraying conditions were found to be 25 MPa and 67 °C [18]. Based on CFD, simulate the ethanol nozzle at a temperature of 360 K, numerical studies have shown that environmental conditions such as temperature can affect the quality and performance of liquid jets, which is difficult to observe in experimental work [19]. CFD analysis was used to verify the influence of back pressure on the spray process of UAV, SOLIDWORKS2017 was used for modeling in the study, and SST k−ε model was selected to describe the turbulence of computational regions, which is suitable for complex shear stress flow. Spatial atomization characteristics were highly correlated with airflow and back pressure. As crosswind generated droplet drift and atomization stratification and downwash could improve the uniformity of droplet distribution, spraying performance was superior by enhancing downwash to restrain the adverse effect of crosswind in real applications [20]. Study the droplet behavior in a droplet ultrasonic atomization reactor using a discrete phase model (DPM) model the results showed that droplet collision and breakage would increase the droplet size, broaden the droplet size distribution and hinder the transport of droplets [21]. The solid protein was coated by the scCO_2_ spraying process when exposed to water or phosphate buffered saline, holding promise for future production of controlled drug delivery systems for therapeutic proteins [22]. The effect of pressure and saturation temperature on the size of scCO_2_ and polylactic acid (PLA) mixed droplets was studied, in situ observation of the viscosity of the plasticized polymer indicates that a backpressure of 68 bar or greater is necessary to ensure the production of fine particles [23]. However, most studies in the past focused on the effect of spraying formula and process on spraying effect and lack of in-depth research on mechanism. Therefore, it is very important to further study the hydrodynamic behavior of the fluid in the spray process to reveal the atomization mechanism of supercritical fluid spray and the effect of relevant process parameters on the atomization effect. The spray characteristics of atomizing nozzles are difficult to determine by experimental measurements, resulting in the spray characteristics based on supercritical fluid spraying not being reported [24].

Computer numerical simulation is an important tool for solving problems that are difficult to solve by traditional experimental methods. Computer numerical simulation has many advantages such as fast speed, low cost, and rich conclusions, etc. Computational fluid dynamics (CFD) simulation is an important tool as it enables engineers to study different design options without a time-consuming experimental workload [25]. Numerical simulation based on computational fluid dynamics is a method of solving fluid dynamics by using computer technology, and all fluid flows abide by the laws of conservation of mass, conservation of momentum, and energy conservation. According to these three laws, the continuity equation, momentum equation, and energy equation can be derived to form the N-S equation. CFD is used to replace the continuous physical field with a collection of values of field variables at a finite number of discrete points and then to obtain the approximate values of the field variables according to the solution of the N-S equation set. At present, computer numerical simulation based on CFD has been widely used in the engineering field to solve difficult problems in practical applications, for example the simulation of macroscopic combustion of PU in a fire [9]. The frictional resistance of polymer coatings is calculated by combining dissipative particle dynamics with CFD [26]. The effect of spraying parameters such as spraying angle on the results in the cold spray deposition process has been studied [27].

In this study, we numerically simulated the spraying process using the commercial software package ANSYS FLUENT 16.0 based on the actual process and parameters of scCO_2_ spraying polyurethane coatings. The flow characteristics inside the tube and the atomization effect outside the tube during the spraying process were investigated with the volume fraction (the ratio of the scCO_2_ volume to the total volume) of scCO_2_ and the inlet pressure.

## 2. Experimental Section

**Figure 1:**
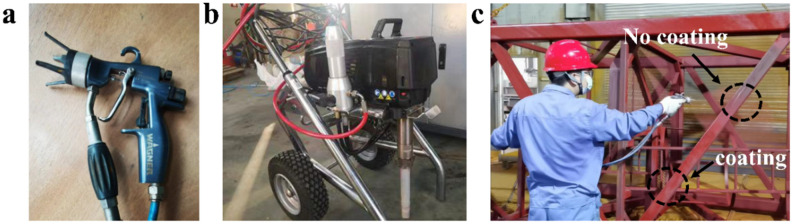
(**a**) Spray gun, (**b**) spraying equipment, and (**c**) spraying construction of polyurethane coating on the steel surface.

Shaoxing Huachuang Polyurethane Co., Ltd. (Shaoxing, China) and Nanjing Forestry University jointly developed the scCO_2_ spraying equipment. The gun and equipment are shown in Figure 1a,b. The spraying equipment has been successfully used to spray PU coatings on the warm exterior walls of livestock holdings, homes, a cold storage floor, etc. The spraying construction of the PU coating on steel surfaces is shown in Figure 1c. The viscosity requirements of the polyurethane coating are quite lenient, the volume fraction of scCO_2_ does not exceed 0.4, the spraying pressure is between 10~25 MPa, and the temperature is between 35~60 °C, ensuring that the CO_2_ is in a supercritical state. The quality of the PU coating was tested according to the HG/T 2454-2014 standard, and the test results indicate that the properties of PU coating are good, as listed in Table 1 [28].

## 3. Numerical Methodology

The 309-type nozzle was modeled based on the experimental basis, i.e., atomization angle of 30° and nozzle diameter of 0.09 inch. The parameters involved in the simulation are listed in Table 2. The volume fraction of scCO_2_ is defined as the volume fraction of supercritical carbon dioxide divided by the total volume of the mixture.

### 3.1. Geometry and Computational Domains

**Figure 2:**
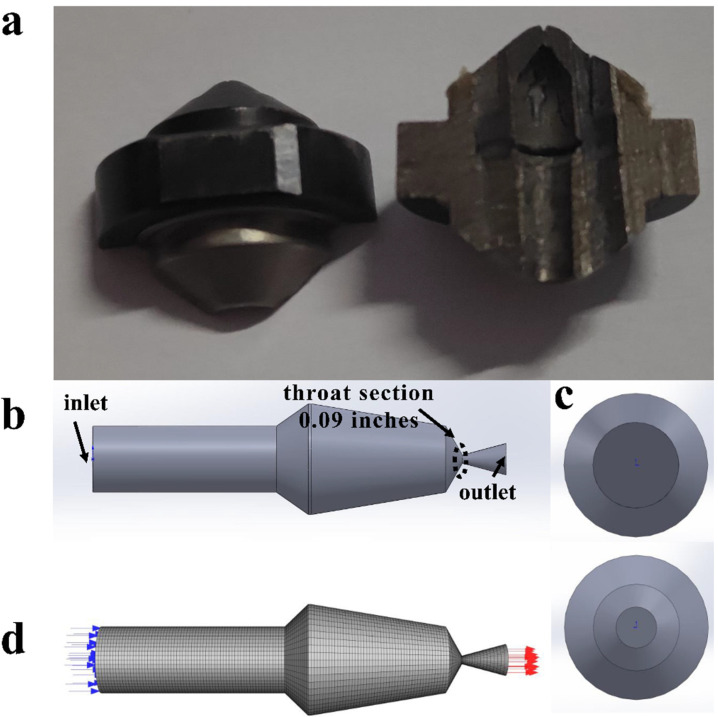
(**a**) Photos of the nozzle used in the experiment. (**b**) Internal fluid domain of the nozzle and the blue and red arrows represent inflow and outflow, respectively. (**c**) Inlet (above) and outlet (below) of the nozzle. (**d**) Grid division of the fluid domain.

Numerical simulation of nozzle flow was carried out using ANSYS-FLUENT V20.2 software to investigate the flow characteristics of atomized discrete droplets in a continuous phase. Figure 2a shows the internal photograph of the nozzle used in the experiment; this nozzle was purchased from Wagner International AG. As shown in Figure 2b, the fluid domain inside the nozzle was 3D modeled using SOLIDWORKS 2017 software with a nozzle outlet diameter of 0.09 inches. Liquid enters the nozzle from the inlet (upper part of Figure 2c) and enters the air from the outlet (bottom part of Figure 2c) through the nozzle. As shown in Figure 2d, the fluid domain inside the nozzle was meshed using an unstructured grid of 70,224 cells and 74,555 nodes. The nozzle exterior was also meshed using an unstructured grid with 128,590 cells and 25,342 nodes. The effect of gravity on the paint is ignored. The nozzle exterior was meshed using a velocity inlet, and the inlet velocity was the exit velocity obtained by solving for the nozzle interior [29]. The steady-state pressure solver is used in this study for the fluid inside the nozzle because it takes into account the compressibility effect and has a good convergence in supersonic cold spray research [30,31,32]. The pressure–velocity couplings are in Couple format, and pressure is in Second Order. Momentum, energy, turbulent kinetic energy, and turbulent dissipation rate are in Second Order Upwind. For the exterior of the nozzle, a transient pressure solver is used. The pressure–velocity couplings are in SIMPLE format, and the Second Order Upwind is used for pressure, momentum, and energy. The First Order Upwind is used for turbulence kinetic energy and turbulence dissipation rate. Atomization occurs in the vicinity of the nozzle, which is rapidly decomposed into droplets by the jet [33]. The particle atomization basin outside the nozzle begins at the throat section of the nozzle and ends 100 mm from the nozzle outlet cross section. The subtle inhomogeneity of fluid velocity in the throat section was ignored, and the atomization effect of the paint was observed after it left the nozzle. Pressure and volume fraction of scCO_2_ were used as inlet boundary conditions. An adiabatic wall with no-slip boundary conditions was used over the entire side surface of the nozzle.

**Figure 3:**
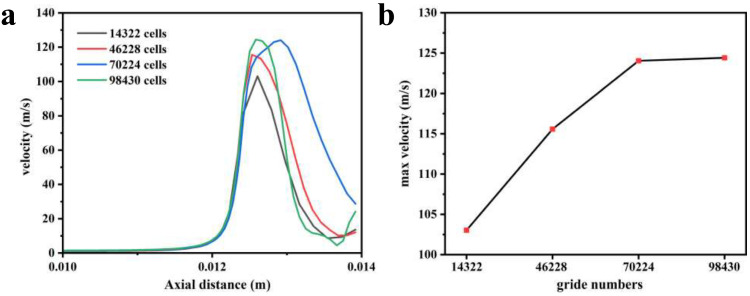
(**a**) The influence of grid number on the axial velocity inside the nozzle; (**b**) maximum internal velocity of nozzles with different grid numbers.

As shown in Figure 3a,b, the number of grids increased from 14,322 to 46,228, and the maximum velocity inside the nozzle increased from 103 m/s to 115 m/s, with a change rate of 11.6%. When the number of grids increases to 70,224, the maximum speed is 124.05 m/s, and the rate of change is 8.7%. When the number of grids reaches 98,430, the maximum speed is 124.4 m/s and the rate of change is 0.2%. Increasing the number of grids further has little effect on the maximum velocity change of the fluid when the number of grids reaches 70,224. For the comprehensive consideration of accuracy and calculation speed, a model with 70,224 grid numbers is selected for calculation.

### 3.2. Numerical Calculation Method

Physicochemical properties of scCO_2_: Above the critical temperature and pressure, the thermodynamic properties of the gas have deviated from those of the ideal gas, and the ideal gas equation of state is no longer applicable. In this study, we derived the density, viscosity, thermal conductivity, and heat capacity of CO_2_ at different pressures and temperatures from the refrigerant physical properties query software REFPROP Version 9.1 and called it through the User-Define (UDF) file.

The PU resin is considered an incompressible fluid.

Polyurethane coating (Hempathane HS 55610) was purchased from Hempel (China) Co., Ltd. (Shanghai, China). The density of the product information table can be queried. The viscosity, thermal conductivity, and specific heat capacity were measured experimentally. The viscosity was measured by using a DV-III+ rheometer produced by Brookfield (China) Co., Ltd(Suzhou, China), at a speed of 12 RPM. The specific heat capacity was measured by DSC sapphire method, model NETZSCH DSC214 Polyma, the heating rate is 5 K/min. The thermal conductivity was measured by Hot Disk TPS 2500S, the heating power is 30 MW and the heating time is 5 s. The physical properties of the coating are as follows: density of 1160 kg/m^3^, viscosity of 2.5 Pa/s, thermal conductivity of 0.054 W/mK, and heat capacity of 1.2 J/(g K). These values are constants in the numerical simulation process.

The turbulence is modeled using a realizable k-ε turbulence model with enhanced wall treatment as it is widely accepted in cold spraying studies and accurately captures the compression effects of scCO_2_ as a compressible fluid during flow [34,35].

(1) $$I=0.16{/Re}^{\frac{1}{8}}$$

where *I* is the turbulence intensity and *Re* is the Reynolds number. The Reynolds number in the system is about 100,000, so the turbulence intensity is set to 5%. The turbulent viscosity ratio usually does not require precise calculation and is typically between 1–10. For moderate turbulence levels, a setting of 10 is sufficient. In this study, we used a default value of 10.

In this study, no chemical reaction occurs between the polyurethane resin and scCO_2_, and no solids are present when the resin is dissolved in scCO_2_. Droplet fragmentation and impact mechanisms (i.e., splashing, diffusion, rebound, and adhesion) are beyond the scope of this study. Furthermore, the collisions between droplets were ignored in the simulations. The volume fraction (the ratio of the resin volume to the total volume) of the droplets is less than 10% when the droplets enter the air, which makes this study applicable to the DPM [36]. There are two types of droplet fragmentation models: the Taylor Analogical Behavior (TAB) model and the fluctuating fragmentation (Wave) model. The TAB model is suitable for the case of low-Weber number jet atomization as well as for the case of low-velocity jets entering into the scalar air. For Weber numbers greater than 100, the Wave model is more suitable. Therefore, the Wave model is used in this study. The basic formula of the WAVE model is shown in Equations (2)–(4). This model considers that the velocity difference between the gas and liquid causes the fragmentation of the jet droplets [36]. In this study, the Discrete Random Walk (DRW) model is used, which applies a stochastic approach to consider the effect of instantaneous turbulent velocity on the particle orbits. Furthermore, the DRW model can comprehensively take into account all aspects of the particle forces, particle turbulence pulsations, and bidirectional coupling between the two phases.

(2) $$\tau =\frac{3.726B_{1}a}{\Lambda \Omega }$$

(3) $$\Lambda =9.02\frac{(1+0.45{Oh}^{0.5})(1+0.4Ta^{0.7})a}{{\left(1+0.87{We}^{1.67}\right)}^{0.6}}$$

(4) $$\Omega \sqrt{\frac{\rho_{1}a^{3}}{\sigma }}=\frac{0.34+0.38{We}^{1.5}}{\left(1+Oh)(1+1.4Ta^{0.6}\right)}$$

(5) $$We=\frac{\rho v^{2}l}{\sigma }$$

Here, *τ* is break-up time, *B_1_* is adjustment parameter in the WAVE breakup model, values of *B*_1_, depending on the injector characterization. *ρ_1_* is the density of polyurethane coatings; *a* is liquid jet radius; *Λ* is the corresponding wavelength of the maximum growth rate; *Ω* is maximum growth rate; *Oh* is the Ohnesorge number; *Ta* is Taylor parameter, *Ta= Oh*$\sqrt{{We}_{g}}$; *We* is weber number; *l* is characteristic length [36,37]. *σ* is the surface tension of the droplet, which was set at 30 mN/m in this study [38].

## 4. Results and Discussions

**Figure 4:**
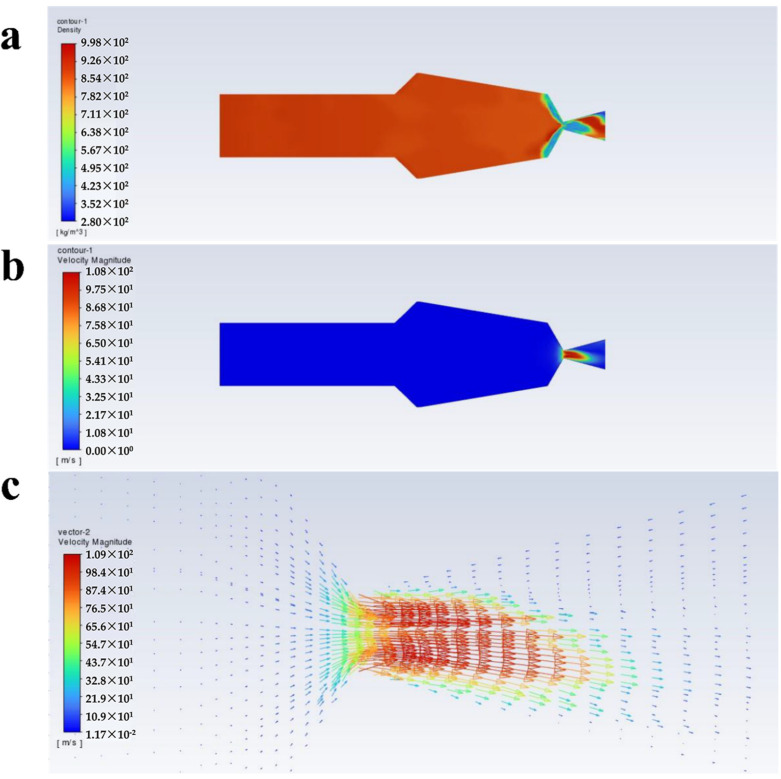
Flow characteristics inside the nozzle at a volume fraction of 0.3 for scCO_2_, and inlet pressure of 10 MPa: (**a**) density cloud, (**b**) velocity cloud, and (**c**) velocity vector plots.

**Figure 5:**
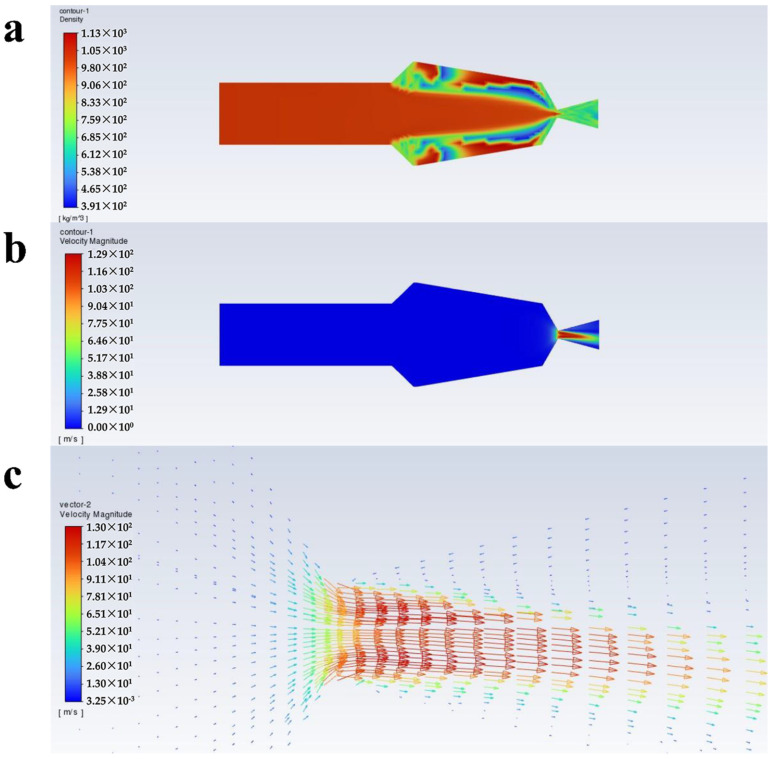
Flow characteristics inside the nozzle at a volume fraction of 0.3 for scCO_2_, and inlet pressure of 20 MPa: (**a**) density cloud, (**b**) velocity cloud, and (**c**) velocity vector plots.

**Figure 6:**
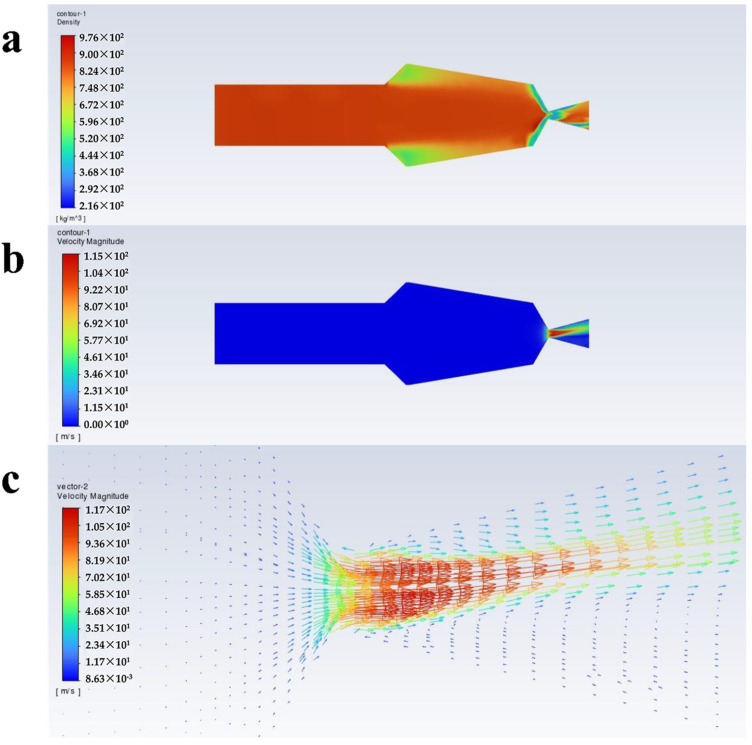
Flow characteristics inside the nozzle at a volume fraction of 0.4 for scCO_2_, and inlet pressure of 10 MPa: (**a**) density cloud, (**b**) velocity cloud, and (**c**) velocity vector plots.

**Figure 7:**
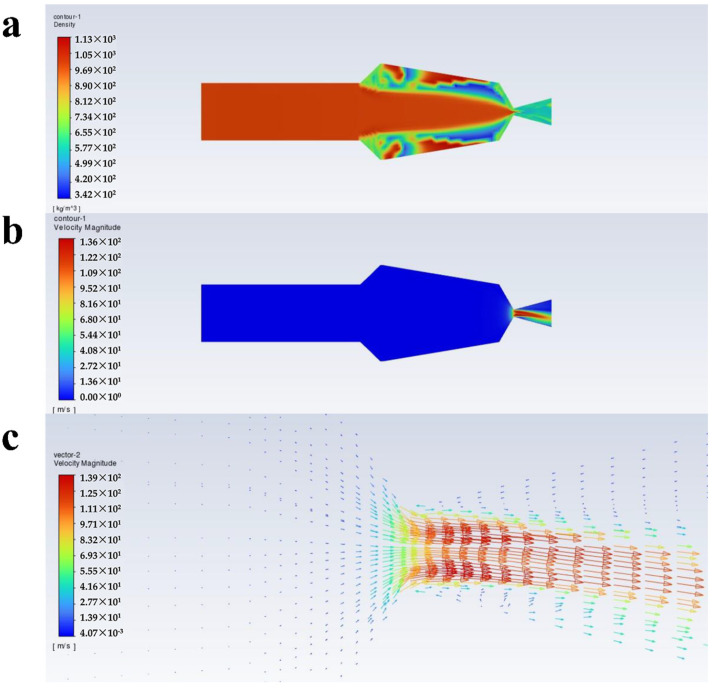
Flow characteristics inside the nozzle at a volume fraction of 0.4 for scCO_2_, and inlet pressure of 20 MPa: (**a**) density cloud, (**b**) velocity cloud, and (**c**) velocity vector plots.

Figure 4, Figure 5, Figure 6 and Figure 7 show the density, velocity cloud, and velocity vector diagram of the nozzle under different conditions (see Table 2). The flow characteristics under four practical engineering application boundary conditions are as follows: the volume fraction of carbon dioxide is 0.3 and 0.4; the inlet pressure is 10 MPa and 20 MPa. In addition, in order to explore the effect of volume fraction on flow characteristics, the flow characteristics of 0.2, 0.5, and 0.6 volume fractions under 10 MPa and 20 MPa inlet pressure are studied (Figures S1–S6). These case calculations all follow similar patterns. Taking case III as an example (Figure 4), the flow characteristics inside the nozzle through the cross section of the nozzle axis are analyzed. As shown in Figure 4a, the density is unchanged when the fluid is in the internal area of nozzle flow. When the fluid passes through the throat section, the pressure decreases dramatically until the nozzle outlet pressure becomes 1 atm, resulting in a decrease in density. The internal area of the nozzle shows non-uniformity of density at the top and bottom sides, which may be due to the phase separation triggered by the fluid moving from the uniform cylindrical basin into the irregular fluid area, and the same situation is also observed in the other three systems. As shown in Figure 4, Figure 5, Figure 6 and Figure 7a, at an inlet pressure of 20 MPa (Figure 5 and Figure 7), the non-uniformity of the density cloud map is more pronounced than at an inlet pressure of 10 MPa (Figure 4 and Figure 6). This is because when the fluid is in the internal region, due to a sudden increase in volume, the fluid density decreases. The closer the fluid microcluster near the wall surface is to the wall, the larger the shear deformation is. The viscous force on the lower surface of the fluid microcluster is larger than that on the upper surface, and the direction of the viscous force is opposite to the direction of the flow, so the movement of the fluid microcluster near the boundary receives obstruction. The scCO_2_ in the fluid microcluster near the boundary generates shear force with the polyurethane resin, so the two liquids move relative to each other, resulting in the separation between the liquid phases. From the density cloud map, it can be seen that the original uniform cloud map exhibits non-uniformity in the internal area. The higher the fluid inlet pressure, the greater the density change, and the more obvious the non-uniformity.

**Figure 8:**
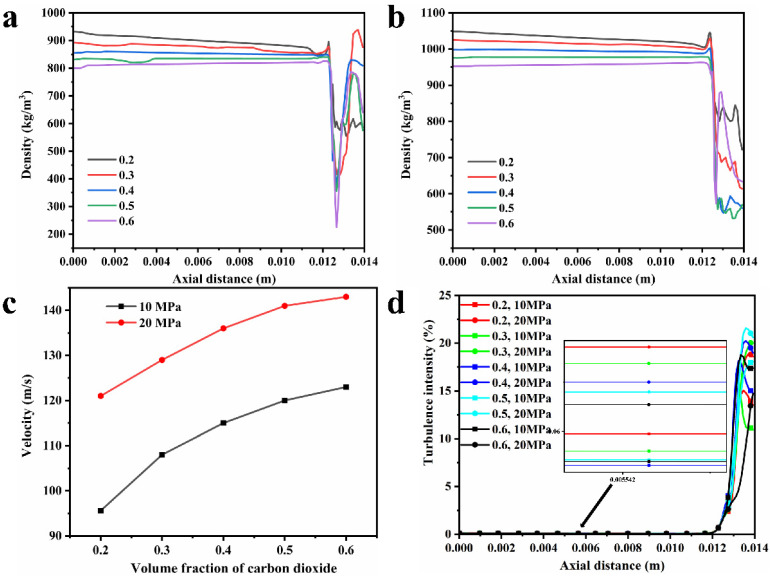
Axial density distribution under inlet pressure of (**a**) 10 MPa and (**b**) 20 MPa, (**c**) velocity of throat section in cases I to X. (**d**) Turbulence intensity in case I to X.

It can be seen from Figure 4b that the velocity varies drastically near the jet port and reaches the maximum value at the throat section. The maximum values of velocity from case I to case X are 95.6, 121, 109, 129, 115, 136, 120, 141, 123, and 143 m/s (Figure 8c), respectively, and the velocity decreases with increasing distance from the throat section. This is caused by the expansion of the air jet and the increase of air resistance [39]. From Figure 4c, it can be seen that from the throat section to the jet port, the velocity appears to have a trend of slowly becoming smaller, which is because the density of scCO_2_ varies with pressure and the hydrostatic pressure rapidly decreases to less than 72.9 atm as it approaches the nozzle. This causes bubbles in the mixture to expand and CO_2_ to transition from a supercritical state to a gaseous state [40]. The density of the coating thus becomes low, which corresponds to Figure 4a. Meanwhile, the decrease in pressure leads to a decrease in velocity. Furthermore, there is no reflux in the outlet section, which facilitates the atomization of the coating with scCO_2_ as a solvent and contributes to the improvement of the spray quality.

**Figure 9:**
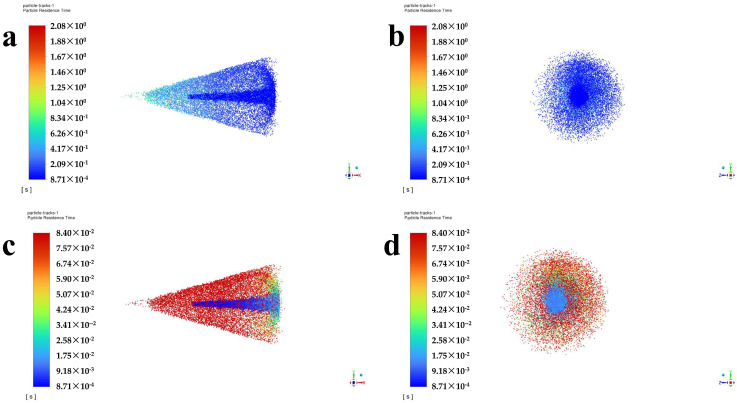
The particle residence time in the cross section passing through the axis (**a**,**c**) and in the plane perpendicular to the axis at a distance of 100 mm from the nozzle (**b**,**d**). The time scales are 0 to 2.08 s in (**a**,**b**), and the time scales are 0 to 0.084 s in (**c**,**d**).

In the four cases, the atomization outside the nozzle also has consistency. Taking case II as an example, Figure 9a,c shows the particle residence time in the cross section passing through the axis, and Figure 9b,d shows the particle residence time in the plane perpendicular to the axis at a distance of 100 mm from the nozzle. The time scales are 0 to 2.08 s in Figure 9a,b, and the time scales are 0 to 0.084 s in Figure 9c,d.

The longest particle residence time reaches 2.08 s. The distribution of particles at each residence time is very uniform. Most of the particles gathered in the middle part with a spray angle of 30°. As shown in Figure 9c,d, by narrowing the time range, it can be observed that the residence time of the discrete phase in the middle aggregation part is slightly smaller than that of the outermost layer, and as the particle trajectory and axis angle increase, the residence time also increases. This is because particles traveling along the axis travel a shorter distance than other particles, so the residence time of the particles is also the shortest.

**Figure 10:**
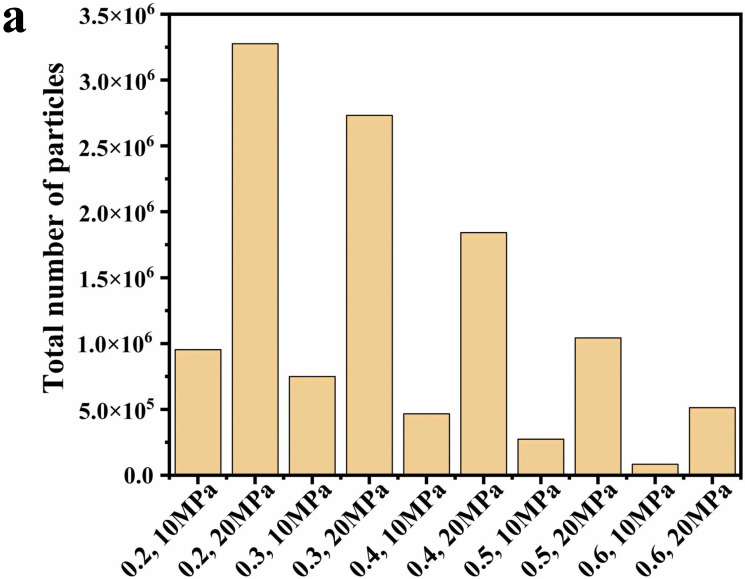

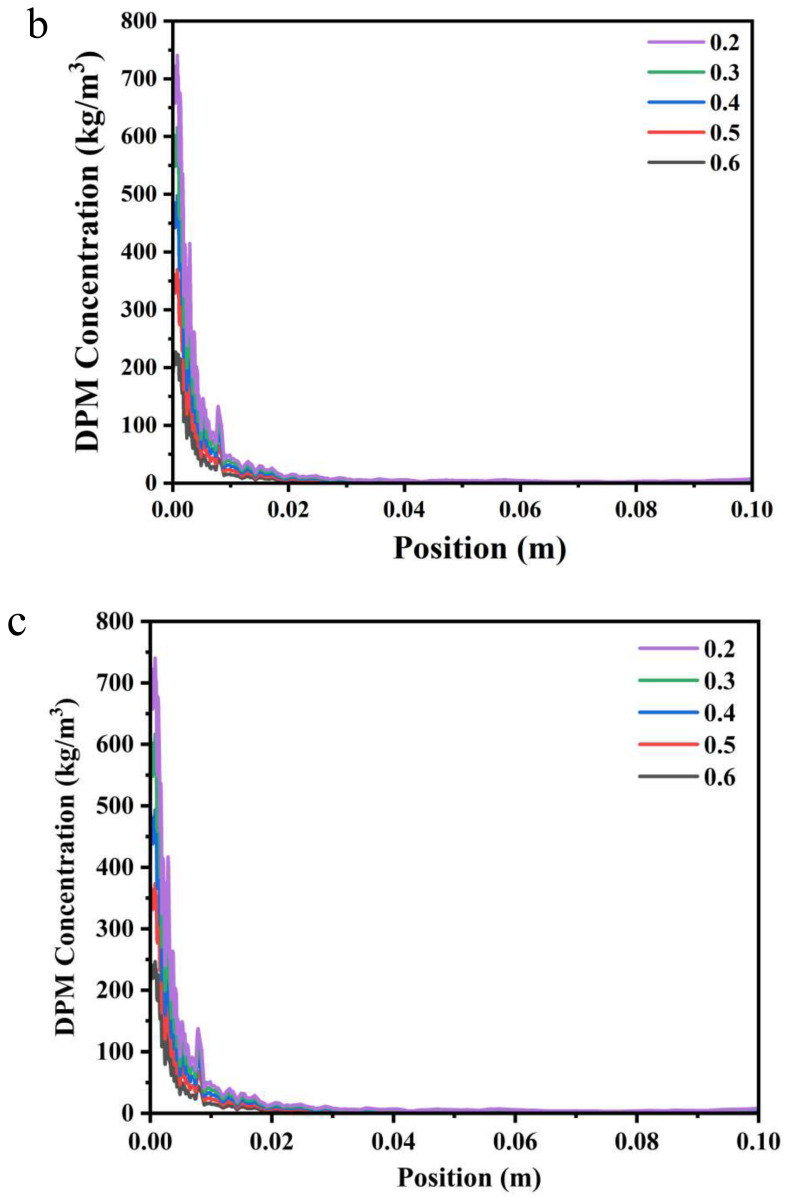
(**a**) Total number of particles in case I to X. Axial DPM concentration distribution under inlet pressure of (**b**) 10 MPa and (**c**) 20 MPa.

Figure 8 demonstrates the effect of inlet pressure and volume fraction of scCO_2_ on the flow characteristics of the coating. Figure 8a–c shows the flow characteristics inside the nozzle. The origin of the *x*-axis is the nozzle inlet out, x = 0.014 m is the spout, and the throat section is located at x = 0.0127 m. As shown in Figure 8a,b, as the volume fraction of carbon dioxide increases from 0.2 to 0.6, the fluid density decreases successively. When the carbon dioxide volume fraction is the same, the fluid density at the inlet pressure of 20 MPa is always greater than the density at the inlet pressure of 10 MPa. The density of scCO_2_ increases with the increase of pressure and reaches the maximum at 20 MPa. The density of scCO_2_ at 20 MPa and 330 K is 840.6 kg/m^3^, which is less than the density of polyurethane coating, and thus the density of the coating and scCO_2_ mixtures becomes smaller when the volume fraction of scCO_2_ increases. When the fluid passes through the throat region, because the air pressure is close to the standard atmospheric pressure, the carbon dioxide is no longer in a supercritical state, and the fluid density will be sharply reduced because the carbon dioxide is transformed into a gaseous state. This is consistent with the density cloud plot (Figure 4, Figure 5, Figure 6, Figure 7 and Figures S1–S6). The velocity is constant when the fluid is in the internal area of the nozzle, and the velocity reaches a maximum value when the fluid passes through the throat section. The two cases with an inlet pressure of 20 MPa have the greatest velocity of the fluid into the air. This is because the greater pressure gives the fluid a greater driving force, and the case with a large volume fraction of scCO_2_ has a greater velocity because the paint density is greater than the scCO_2_. The nozzle is prone to clogging when the volume fraction of the coating increases [41]. In conjunction with Figure 8c, a fluid with a large volume fraction of scCO_2_ will obtain a smaller density, resulting in a larger velocity, and a larger velocity also results in a larger distance for the spray to effectively spray. The closer to the nozzle, the closer the pressure is to atmospheric pressure, and the CO_2_ is also no longer in a supercritical state at this time, resulting in the flow characteristics of internal fluid changing considerably. In Figure 8d, there is a sharp increase in the intensity of turbulence through the throat region, which also leads to a decrease in velocity. In addition, the turbulence intensity at 20 MPa inlet pressure is always greater than that at 10 MPa inlet pressure. In Figure 10b,c, the throat section of the nozzle is at x = 0 and the spout is at x = 0.0015 m. When x exceeds 0.0015 m, this means that the fluid enters the air. It is clear that systems with a higher polyurethane coating content have a higher DPM concentration at atomization. For the same volume fraction, the inlet pressure of 20 MPa will make the DPM concentration slightly larger than the DPM concentration at 10 MPa, because the larger inlet pressure corresponds to the larger initial velocity on the axis inside the nozzle. In the case of the same outlet area and fluid physical properties, a larger speed will lead to a larger mass flow rate outside the nozzle. Higher coating content and smaller inlet pressures will result in higher DPM concentrations, and thus a smaller inlet pressure should be used to make the droplets more uniform across the 30° spray range. Figure 10a shows the total number of droplet particles under different boundary conditions. As the content of polyurethane coatings decreases, the particle number also decreases. In the case of the same volume fraction, a larger inlet pressure will increase the total number of droplets, that is, the atomization effect is better. This phenomenon is also verified in Table 3, where greater inlet pressure for the same volume fraction leads to greater movement speed, which makes the droplet size smaller.

## 5. Conclusions

To investigate the flow characteristics of coatings with scCO_2_ as a solvent during the actual production process, a systematic study was carried out by numerical simulation. The effects of inlet pressure and volume fraction of scCO_2_ on the fluid motion parameters inside the nozzle as well as the atomization effect of droplets outside the nozzle were investigated. The following conclusions can be drawn:

1. At an inlet pressure of 10 MPa, as the volume fraction of scCO_2_ increases from 0.2 to 0.6, the velocity of the paint through the throat section is 95.6 m/s, 108 m/s, 115 m/s, 120 m/s, and 123 m/s. In the other cases with an inlet pressure of 20 MPa, the velocity is 121 m/s, 129 m/s, 136 m/s, 141 m/s and 143 m/s. There is a decreased tendency for the velocity from the throat section to the nozzle outlet. Near the nozzle, the density becomes inhomogeneous and the turbulence intensity increases.
2. The velocity vector plots under the ten conditions do not show any obvious backflow, which is conducive to the improvement of the atomization effect of the paint with scCO_2_ as the solvent, and contributes to improving the spraying quality.
3. At the same inlet pressure, systems with a large scCO_2_ volume percentage have a higher DPM concentration near the shaft compared to coatings with a small scCO_2_ volume fraction. For systems with the same scCO_2_ volume fraction, the DPM concentration at 10 MPa inlet pressure is slightly greater than that at 20 MPa inlet pressure.
4. The higher inlet pressure results in better atomization of the paint, smaller particles, and more quantities. While a larger carbon dioxide volume fraction will further reduce the droplet size, the total number of particles is small because the polyurethane coating content is too low. Based on the consideration of the atomization effect and actual needs, it is recommended to use 0.4 volume fraction and 20 MPa inlet pressure for spray painting in industry.

## Figures and Tables

**Table 1:** Test results of polyurethane coating.

Test Items	Technical Requirements	Test Result
Pencil scratch hardness test	≥F	HB
Bending test (mm)	2	4
Impact resistance (cm)	50	50
Cross cut test (spacing 3 mm), level	≤1	1
Pull-off test (MPa)	≥4	14 (12.1~15.7), 100%B

**Table 2:** Parameters involved in the simulations.

Condition	Case I	Case II	Case III	Case IV	Case V	Case VI	Case VII	Case VIII	Case IX	Case X
Driving gas pressure (MPa)	10	20	10	20	10	20	10	20	10	20
Driving gas temperature (K)	330	330	330	330	330	330	330	330	330	330
Volume fraction of scCO_2_	0.2	0.2	0.3	0.3	0.4	0.4	0.5	0.5	0.6	0.6

**Table 3:** DPM particle diameter size.

	Maximum Particle Diameter, 10^−5^ (m)	Minimum Particle Diameter, 10^−5^ (m)	Overall Mean Diameter, 10^−5^ (m)
Case I	4.3	2.2	3.7
Case II	2.8	1.4	2.3
Case III	3.5	1.7	3.0
Case IV	2.6	1.2	2.1
Case V	3.3	1.5	2.7
Case VI	2.4	1.1	1.9
Case VII	3.1	1.2	2.4
Case VIII	2.4	0.9	1.8
Case IX	3.0	1.2	2.3
Case X	2.3	0.9	1.7

## Data Availability

Data are contained within the article.

## Supplementary Materials

The following supporting information can be downloaded at: https://www.mdpi.com/article/10.3390/polym16070940/s1, Figure S1: Flow characteristics inside the nozzle at a volume fraction of 0.2 for scCO_2_, and inlet pressure of 10 MPa: (a) density cloud, (b) velocity cloud, and (c) velocity vector; Figure S2: Flow characteristics inside the nozzle at a volume fraction of 0.2 for scCO_2_, and inlet pressure of 20 MPa: (a) density cloud, (b) velocity cloud, and (c) velocity vector plots; Figure S3: Flow characteristics inside the nozzle at a volume fraction of 0.5 for scCO_2_, and inlet pressure of 10 MPa: (a) density cloud, (b) velocity cloud, and (c) velocity vector plots; Figure S4: Flow characteristics inside the nozzle at a volume fraction of 0.5 for scCO_2_, and inlet pressure of 20 MPa: (a) density cloud, (b) velocity cloud, and (c) velocity vector plots; Figure S5: Flow characteristics inside the nozzle at a volume fraction of 0.6 for scCO_2_, and inlet pressure of 10 MPa: (a) density cloud, (b) velocity cloud, and (c) velocity vector plots; Figure S6: Flow characteristics inside the nozzle at a volume fraction of 0.6 for scCO_2_, and inlet pressure of 20 MPa: (a) density cloud, (b) velocity cloud, and (c) velocity vector plots.
