# Supplementary material for: Numerical Simulation of Flow Characteristics for Supercritical CO2-Sprayed Polyurethane Resin
Source: Polymers (Basel). 2024 Mar 29;16(7):940. doi: 10.3390/polym16070940 (PMC11013771; doi:10.3390/polym16070940)
Supplement: Supplementary file 1 [file polymers-16-00940-s001.zip › polymers-2893995-supplementary.pdf]

Supplementary Materials:

Numerical simulation of flow characteristics for supercritical CO_2-sprayed polyurethane resin

Chichao Li[1], Chengrui Zhang[1], Minghua Xiang[2], Qing Chen, Zhenyang Luo[1], Yanlong Luo[1,*]

1 College of Science, Nanjing Forestry University, Nanjing 210037, China; l885661983@gmail.com (C.L.); zcr19850410054@163.com (C.Z.); luozhenyang@njfu.edu.cn (Z.L.)

2 Shaoxing Huachuang Polyurethane Co., Ltd., Shaoxing 312037, China; minghuaxiang2024@163.com

3 College of Mechanical and Electronic Engineering, Nanjing Forestry University, Nanjing 210037, China; qchen@njfu.edu.cn

* Correspondence: luoyanlong@njfu.edu.cn

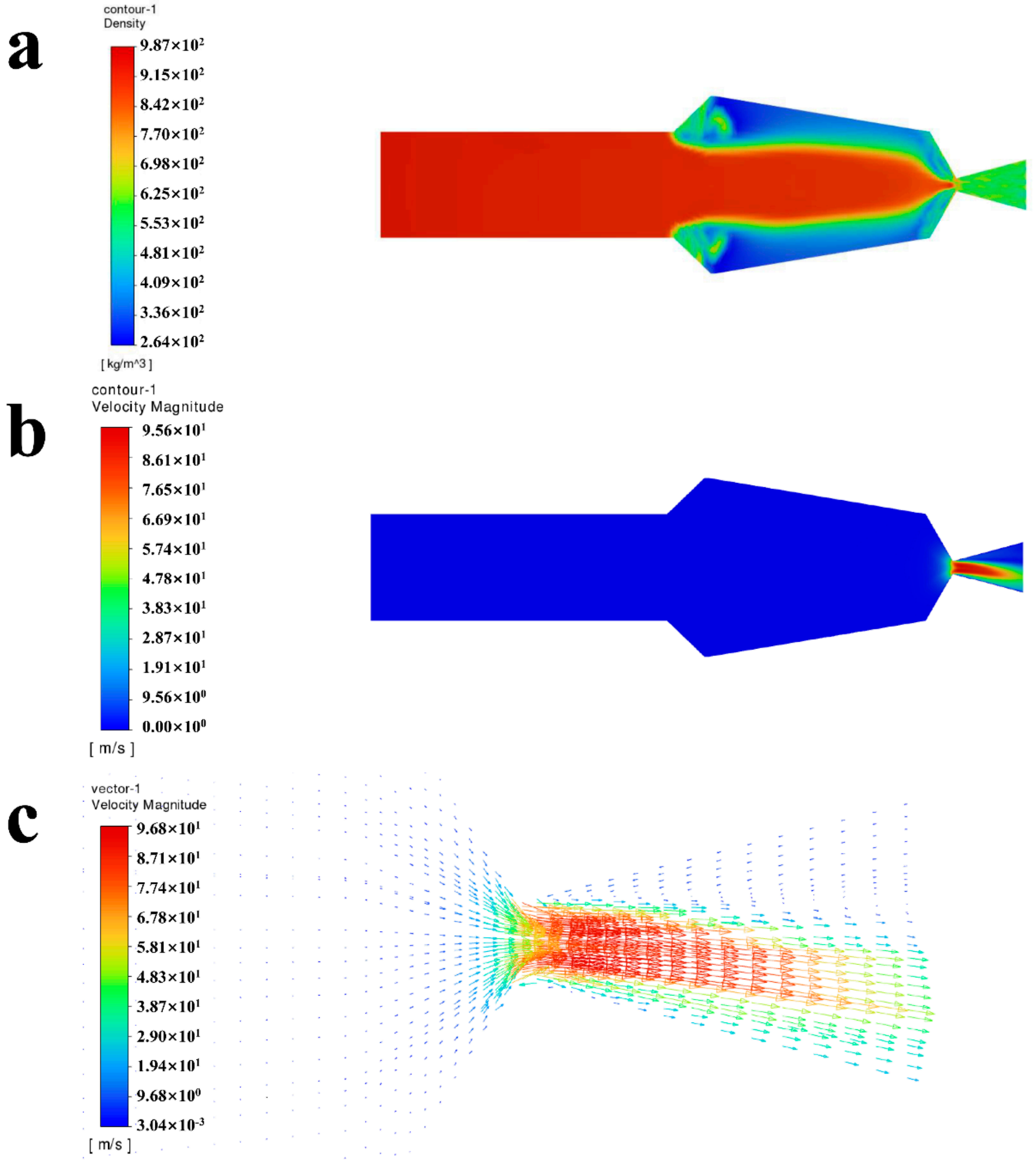

Figure S1 Flow characteristics inside the nozzle at a volume fraction of 0.2 for $scCO_2$,

and inlet pressure of 10 MPa: (a) density cloud, (b) velocity cloud, and (c) velocity vector. plots.

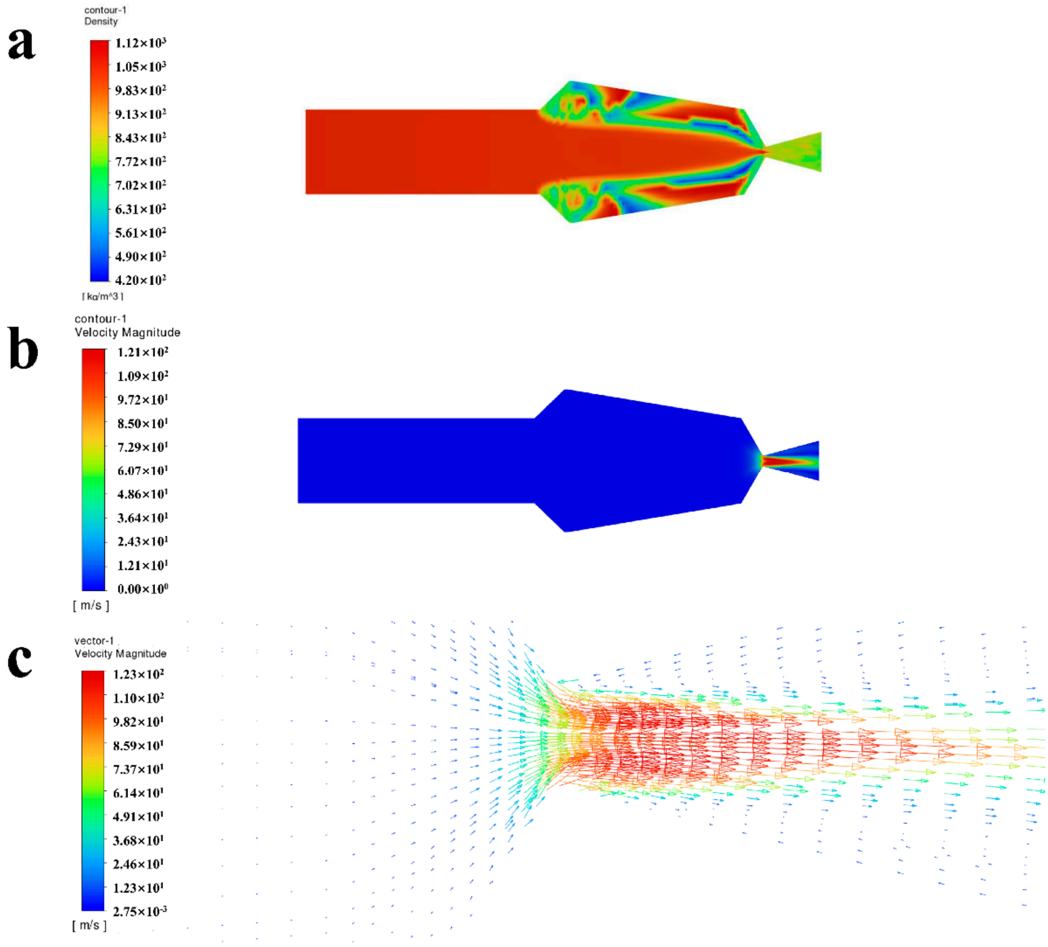

Figure S2 Flow characteristics inside the nozzle at a volume fraction of 0.2 for $scCO_2$, and inlet pressure of 20 MPa: (a) density cloud, (b) velocity cloud, and (c) velocity vector plots.

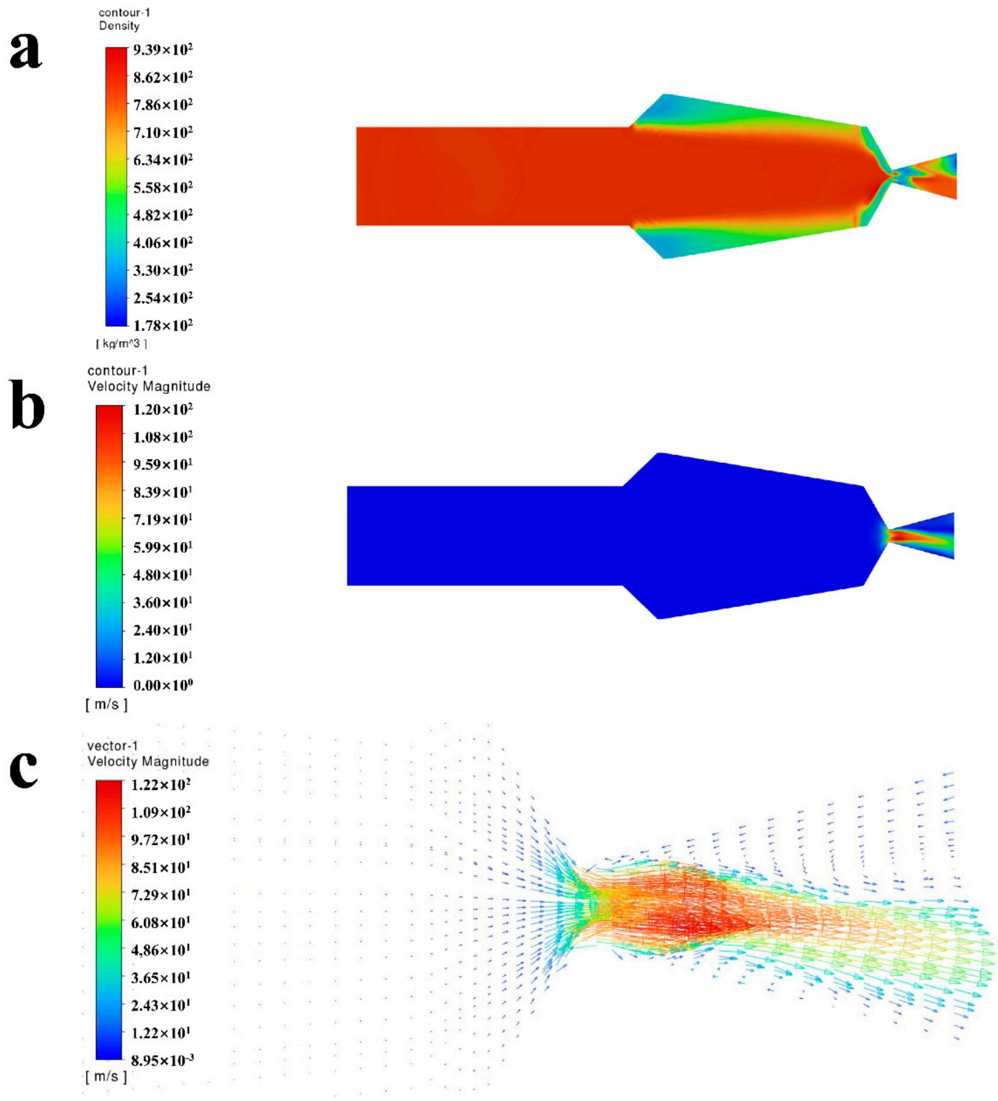

Figure S3 Flow characteristics inside the nozzle at a volume fraction of 0.5 for $scCO_2$, and inlet pressure of 10 MPa: (a) density cloud, (b) velocity cloud, and (c) velocity vector plots.

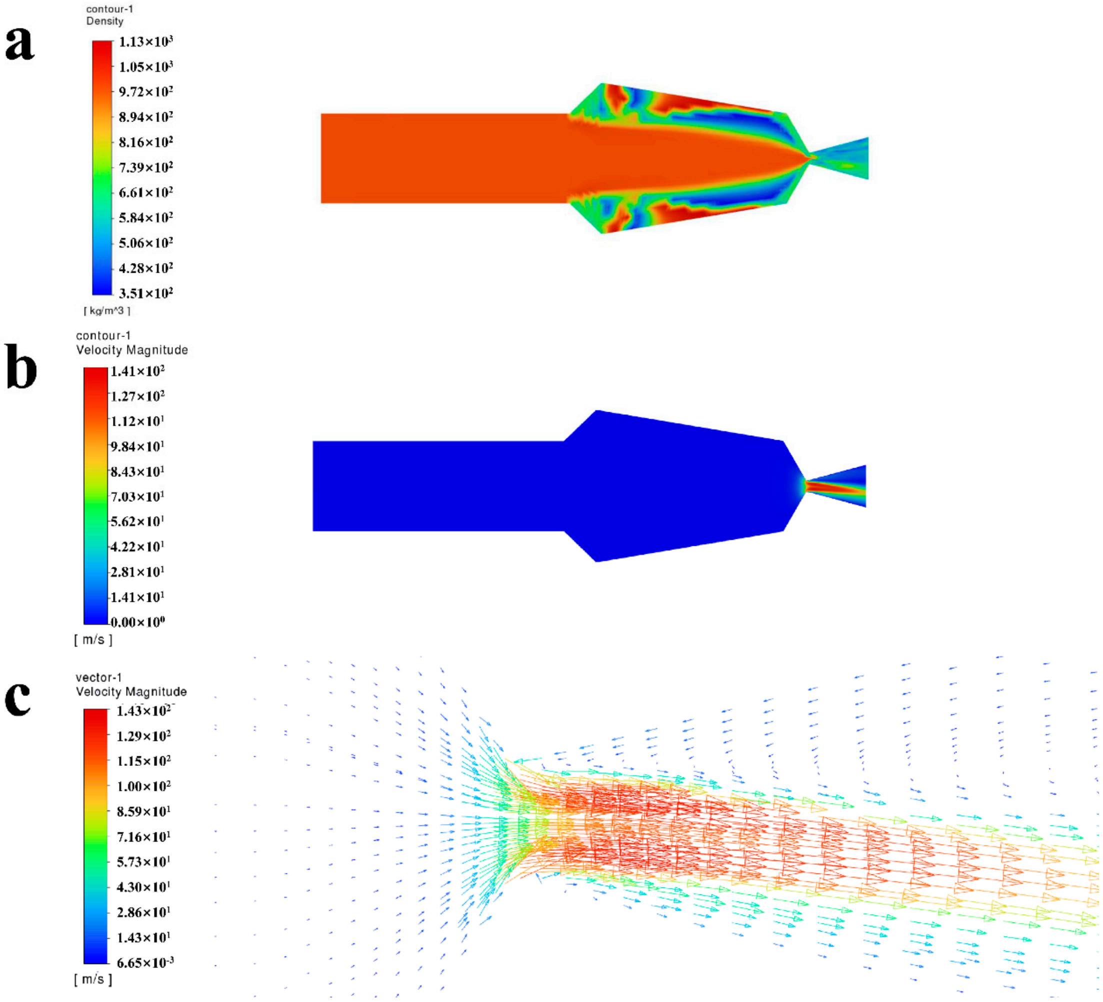

Figure S4 Flow characteristics inside the nozzle at a volume fraction of 0.5 for $scCO_2$, and inlet pressure of 20 MPa: (a) density cloud, (b) velocity cloud, and (c) velocity vector plots.

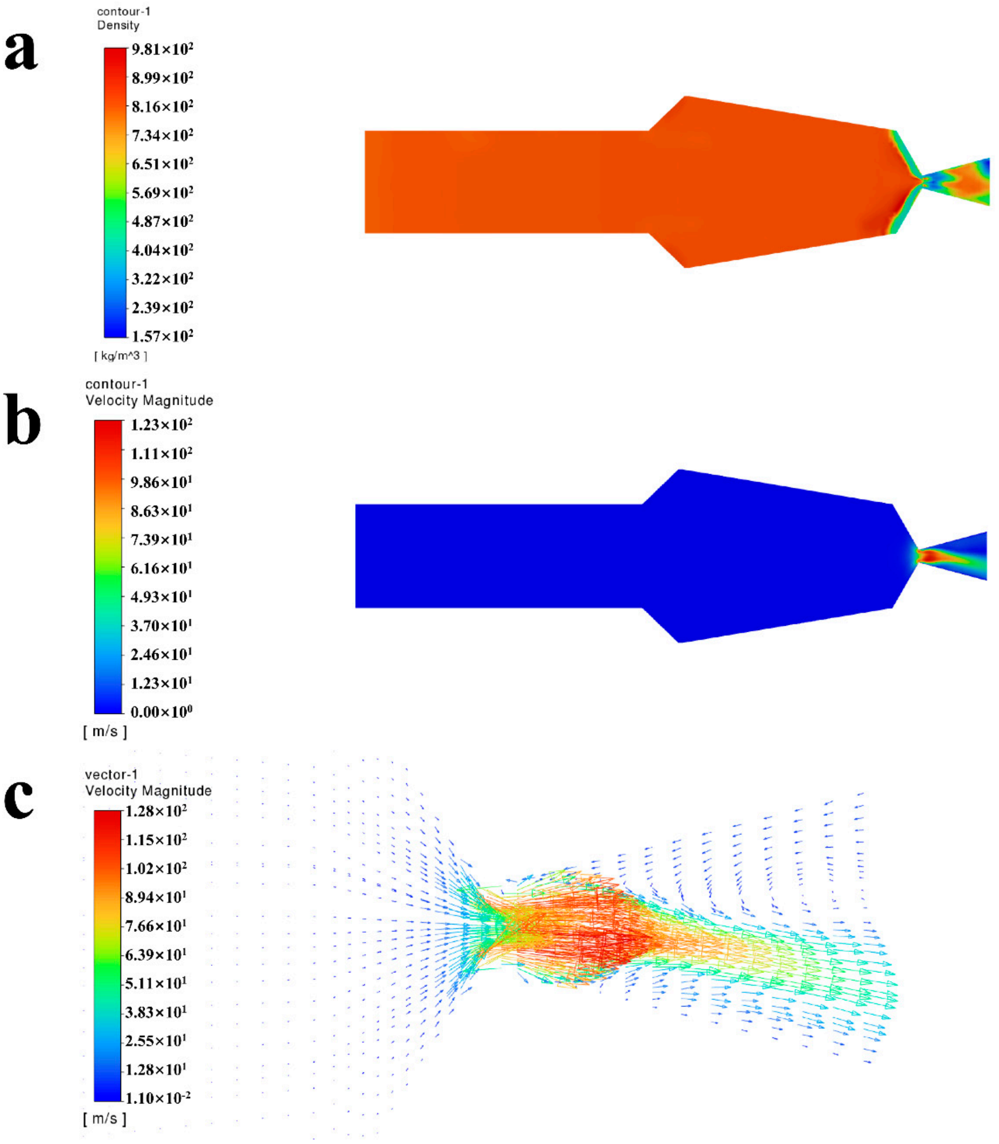

Figure S5 Flow characteristics inside the nozzle at a volume fraction of 0.6 for $scCO_2$, and inlet pressure of 10 MPa: (a) density cloud, (b) velocity cloud, and (c) velocity vector plots.

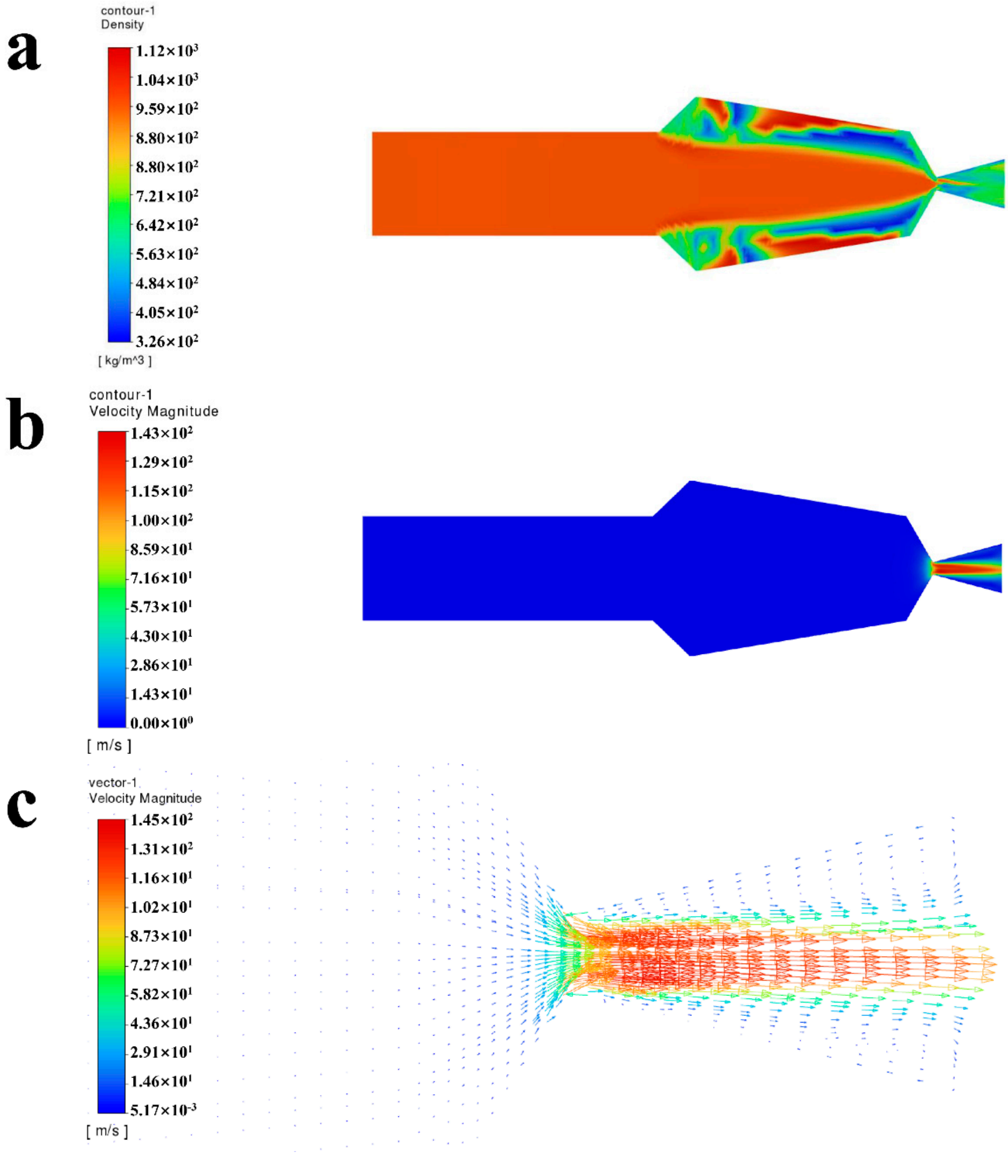

Figure S6 Flow characteristics inside the nozzle at a volume fraction of 0.6 for $scCO_2$, and inlet pressure of 20 MPa: (a) density cloud, (b) velocity cloud, and (c) velocity vector plots.
